# Age‐ and sex‐dependent alterations in primary somatosensory cortex neuronal calcium network dynamics during locomotion

**DOI:** 10.1111/acel.13898

**Published:** 2023-06-03

**Authors:** Sami L. Case, Ruei‐Lung Lin, Olivier Thibault

**Affiliations:** ^1^ Department of Pharmacology & Nutritional Sciences University of Kentucky College of Medicine Lexington Kentucky USA

**Keywords:** behavior, locomotor stability, mice, neuronal network, neuroscience, Ca^2+^, somatosensation, two‐photon imaging

## Abstract

Over the past 30 years, the calcium (Ca^2+^) hypothesis of brain aging has provided clear evidence that hippocampal neuronal Ca^2+^ dysregulation is a key biomarker of aging. Age‐dependent Ca^2+^‐mediated changes in intrinsic excitability, synaptic plasticity, and activity have helped identify some of the mechanisms engaged in memory and cognitive decline based on work done mostly at the single‐cell level and in the slice preparation. Recently, our lab identified age‐ and Ca^2+^‐related neuronal network dysregulation in the cortex of the anesthetized animal. Still, investigations in the awake animal are needed to test the generalizability of the Ca^2+^ hypothesis of brain aging. Here, we used *in vigilo* two‐photon imaging in ambulating mice, to image GCaMP8f in the primary somatosensory cortex (S1), during ambulation and at rest. We investigated aging‐ and sex‐related changes in neuronal networks in the C56BL/6J mouse. Following imaging, gait behavior was characterized to test for changes in locomotor stability. During ambulation, in both young adult and aged mice, an increase in network connectivity and synchronicity was noted. An age‐dependent increase in synchronicity was seen in ambulating aged males only. Additionally, females displayed increases in the number of active neurons, Ca^2+^ transients, and neuronal activity compared to males, particularly during ambulation. These results suggest S1 Ca^2+^ dynamics and network synchronicity are likely contributors of locomotor stability. We believe this work raises awareness of age‐ and sex‐dependent alterations in S1 neuronal networks, perhaps underlying the increase in falls with age.

## Graphical abstract

Here, we used *in vigilo* two‐photon imaging in ambulating mice across age and sex, to image GCaMP8f in the primary somatosensory cortex (S1) and characterize neuronal networks. During ambulation, in both young adult and aged mice, an increase in network connectivity and synchronicity was noted. An age‐dependent increase in synchronicity was seen in ambulating aged males only. Additionally, females displayed increases in the number of active neurons, Ca^2+^ transients, and neuronal activity compared to males, particularly during ambulation.

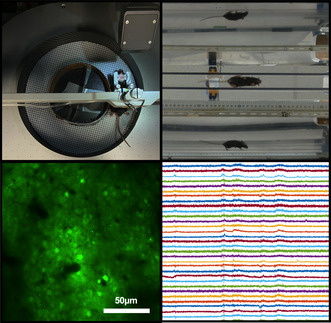

**Abbreviations**
2P: two‐photon
AAV: adeno‐associated virus
Ca2+: calcium
CC: correlation coefficient
CWT: continuous wavelet transform
EEG: electroencephalogram
FOV: field(s) of view
L‐VGCC: L‐type voltage‐gated calcium channel
RM: repeated measures
ROI: region of interest
RyR: ryanodine receptor
S1: primary somatosensory cortex

## 1 INTRODUCTION

The calcium (Ca^2+^) hypothesis of brain aging is still considered critical, influential, and viable and neuronal Ca^2+^ dysregulation has been recognized for over 30 years as a key biomarker of aging (Khachaturian, 1989; Landfield, 1987). The importance of Ca^2+^ in the brain extends back to the early work of Kostyuk and colleagues describing the presence of voltage‐gated Ca^2+^ channels in the plasma membrane of snail neurons (Kostyuk et al., 1977), which was later confirmed in human skin fibroblasts using radioactive Ca^2+^‐labeling (Peterson et al., 1985). Around that time, many others were characterizing Ca^2+^ processes, including kinetics of Ca^2+^‐ATPases (Michaelis et al., 1984), Ca^2+^ currents (Landfield, 1987), and intracellular Ca^2+^ stores (Murchison & Griffith, 1999), conducting the work in culture and rodent models of aging and Alzheimer's disease. Together, these studies helped frame the “Ca^2+^ hypothesis of brain aging”. Details that emerged early during this critical period highlighted the importance of several Ca^2+^‐sensitive proteins that could contribute to alterations in neurotransmission, membrane excitability, and synaptic plasticity (Miller, 1991; Verkhratsky, 2005). However, there are prominent limitations that have hindered a more recent expansion of the Ca^2+^ hypothesis of brain aging including: (1) a restricted network evaluation based on unicellular assessments, (2) the use of ex vivo preparations, and (3) a focus on hippocampal‐centric approaches.

While synaptic communication has been well‐characterized electrophysiologically in specific brain regions in limited numbers of neurons across age, these techniques may not be able to predict neuronal network variables. Subsets of neurons within these neuronal networks work together to perform a specific function or to encode information, such as sensation or memory. These neurons are known to communicate as an “ensemble”, where individual neurons can report on limited information but when acting together in a network, can provide a rich characterization of the environment or context (Aery Jones & Giocomo, 2023). Neuronal ensembles can be defined based on their activity patterns, connectivity, and synchronicity (Carrillo‐Reid & Yuste, 2020). These ensembles are dynamic, reflective of changes over time, and alterations in recruitment depends on the task, the context, and the state of the subject (Guzowski et al., 1999; Lacagnina et al., 2019; Ryan et al., 2015). Of note, a majority of the literature on brain aging, including studies on neuronal ensembles, has focused on cognitive processes related to hippocampal functions of memory and learning (Guzowski et al., 1999; Lacagnina et al., 2019; Reijmers et al., 2007; Ryan et al., 2015), as these tend to be critically modified behaviors with both normal aging and aging‐related neurodegenerative diseases. Furthermore, it is clear that other processes, such as gait behavior, are also altered with age, where ambulation patterns become irregular and slower. Given that gait dysregulation is highly correlated with increased incidence of falls in the older adult population (Krauss et al., 2005), further investigations focused on central components of ambulation are warranted.

Gait reflects on the pattern in which a person ambulates and is known to change with age and neurological status (Beauchet et al., 2016), and in some cases, can lead to an increased risk of falls and fall‐related deaths. Common changes in ambulation patterns seen in both humans and animals include slower walking speed, shorter stride length, increased double support time, wider base of support, and reduced knee/ankle flexion (Judge et al., 1996; Kwon et al., 2018). These changes occur under a variety of conditions, where changes in muscle strength, flexibility, and balance, as well as changes in key brain centers that control ambulation and proprioception can contribute to gait dysregulation (Case et al., 2022). Some work has highlighted that neuronal activity in primary sensorimotor cortices appears to be sensitive to aging as well as Ca^2+^ processes. Specifically, many reports have shown hypoexcitability with age in the primary motor cortex, highlighting reduced motor output, perhaps mediated by loss of functioning upper motor neurons (Clark & Taylor, 2011). Changes in neurophysiological and behavioral components pertaining to gait have been shown to be dysregulated with age (Case et al., 2022), such as increases in neuronal excitability and receptive field size in the primary somatosensory cortex (S1) (Hickmott & Dinse, 2013; Popescu et al., 2021; Spengler et al., 1995) as well as decreased tactile acuity (Lenz et al., 2012). Whether these changes are mediated by short‐term excessive levels of neuronal Ca^2+^, or by a more subtle, yet sustained alteration in Ca^2+^ signaling is not clear. Furthermore, many of these experiments were performed at the single‐ or multi‐cell level and may not accurately reflect in vivo neurophysiology, as neurons communicate at the local and whole brain network levels. Thus, it seems critical to investigate central Ca^2+^ dysregulation with age in these key areas.

Here, and for the first time, we used *in vigilo* two‐photon (2P) imaging techniques to measure neuronal Ca^2+^ dynamics at the single‐cell and network levels across ~2000 neurons in S1 of young and aged ambulating mice. Additionally, we investigated all measures in both males and females to elucidate potential sex differences in neuronal network and behavioral phenotypes. In this study, we test the hypothesis that alterations in S1 neuronal networks in ambulating mice impact distinct patterns of gait behavior, and these alterations differ across both age and sex.

## 2 METHODS

### 2.1 Animals

The work presented here strictly adheres to our Institutional Animal Care and Use Committee protocol. On Week 0, young adult (4 months; male *n* = 11, female *n* = 12) and aged (22 months; male *n* = 10, female *n* = 10) C57BL/6J mice were received from The Jackson Laboratory (Bar Harbor, MN). On Week 1, animals underwent adeno‐associated virus (AAV) injection and installation of a chronic cranial window and headplate. During Weeks 2–4, animals were allowed to recover. On Week 5, animals were tested using a grip strength meter to measure forelimb and hind limb grip strengths. On Week 6, animals were habituated to the Neurotar Mobile HomeCage and 2P room and trained to ambulate during head‐fixation. On Week 7, following 6 weeks of GCaMP8f expression, 2P neuronal Ca^2+^ imaging in S1 cortex was obtained. On Week 8, gait parameters were obtained while animals ambulated on the 3‐plane visualization walking task apparatus.

### 2.2 AAV injection and chronic cranial window surgery

After 1 week of acclimation to vivarium conditions, animals received aseptic injections of AAV (1:1 dilution of 1 × 10^13^ vg/mL stock in sterile saline) carrying the neuron‐specific Ca^2+^ indicator GCaMP8f (pGP‐AAV‐syn‐jGCaMP8f‐WPRE; Addgene #162376‐AAV9). Briefly, animals were anesthetized (1.5%–3.5% isoflurane) and placed on a warming pad (37°C), then head‐fixed to a stereotaxic frame (Kopf, Tujunga, CA) using non‐penetrating ear bars. Artificial tears (GenTeal®) were placed on each eye and 0.5 mL warm sterile saline was administered subcutaneously. PhysioSuite (Kent Scientific Corporation) was used to monitor physiological outcomes (heart and respiratory rate, body temperature, and O_2_ saturation) and to control the warming pad. Hair above the skull was wetted with an alcohol pad, then shaved using a sterile scalpel. After application of alcohol and povidone‐iodine to the shaved skin, a circular piece of tissue was removed around the boundaries of the scalp to expose the skull. A motorized hand drill (Foredom K. 1070 High Speed Rotary Micromotor Kit; Blackstone Industries, Bethel, CT) was then used to remove the periosteum from the bone, followed by application of acetone to degrease the skull. Bonding agent (VivaPen®; Ivoclar Vivadent, Schaan, Liechtenstein) was applied to cover the skull and cured by UV light (LY‐B200 Dental LED Curing Light). A 3‐mm diameter hole was then drilled stereotaxically (Drill and Injection Robot with automatic depth detection; Neurostar, Tubingen, Germany) on either the left or right skull directly above S1 (center of 3‐mm hole: ML = ±1.62 mm; AP = −0.5 mm). The space above the brain was irrigated with cold saline following removal of the 3‐mm bone flap. Advancement/retraction speed of the drill was set to 1.0 mm/min. AAV injection (0.25 μL at 0.2 μL/min) was accomplished (ML = ±1.62 mm; AP = −0.5 mm; DV = −0.40 mm) using a Hamilton® syringe with a 30° bevel. A 4‐mm circular glass coverslip (CS‐4R; Warner Instruments, Holliston, MA) and stainless steel headplate (Model 5 Headplate; Neurotar, Helsinki, Finland) were attached to the bone using light‐curing dental cement (Fusion Flo; Prevest Denpro Ltd., Jammu, India). Following this, animals received subcutaneous injections of meloxicam (10 mg/kg) and buprenorphine (0.2 mg/kg) and were temporarily placed in a heated cage for recovery. Once fully awake and ambulating normally, animals were returned to their home cage. Postoperative inflammation was controlled by re‐administration of subcutaneous meloxicam (10 mg/kg) 24 h following surgery. Animals recovered for 3 weeks to allow inflammation reduction and adequate expression of GCaMP8f.

### 2.3 Grip strength testing

To test neuromuscular function between age groups, all animals underwent grip strength testing of forelimb and hindlimbs using a digital grip strength meter (Columbus Instruments; Columbus, OH). For forelimb measures, the mouse was lowered over a horizontal grid, allowing only its forepaws to attach, then gently pulled back by the tail until a maximum grip strength value was recorded. For hind limb measures, the mouse was lowered over a grid with a 30°C downward tilt, then measuring the maximum force when the animal pushed off to jump from the grid. For all limbs combined, the mouse was lowered over the horizontal grid, allowing both the forepaws and hindpaws to attach, then gently pulled back by the tail until a maximum grip strength value was recorded. All measures were repeated three times per animal, then averaged to yield one value per measurement per animal. We present grip strength measures collected from 36 GCaMP8f‐treated animals (young male *n* = 8, young female *n* = 11, aged male *n* = 9, aged female *n* = 8).

### 2.4 Neurotar mobile HomeCage environment

To characterize neuronal networks during ambulation across multiple surfaces, animals were trained to ambulate in a floating carbon‐fiber cage environment (Neurotar Mobile HomeCage Large). Six days prior to 2P imaging, animals were habituated to the environment in the 2P imaging room for 2 days, then handled to allow habituation to brief head‐fixation (using fingers to grab headbar for periods of 5 s) for an additional 2 days. Animals then underwent acclimation to 20‐min periods of head‐fixation in the Neurotar environment for 2 days prior to imaging, in which they could navigate the donut maze (Figure S1). Animals that did not ambulate around the donut after a 20‐min period were considered to have failed to adapt to the task and were removed from the study.

### 2.5 2P microscopy

2P imaging was accomplished in layers 2/3 of S1 (~200 μm below the dura) using a Scientifica Hyperscope (Uckfield, United Kingdom) equipped with scanning mirrors (1 resonant, 2 galvos), a large back aperture objective (16X, NA = 0.8; WD = 3.0 mm; Nikon), and a GaAsP detector mounted inside a multiphoton detection chamber (MDUXL) housing dichroic and infrared blocking filters. All hardware and data acquisition were controlled by ScanImage (v2021.0.0; Vidrio Technologies) running under MATLAB (vR2020b; MathWorks, Natick, MA). GCaMP8f was excited at 930 nm using an InSight X3 dual‐wavelength femtosecond‐pulsed laser (Spectra‐Physics). Image acquisition (512 × 512 pixels at 30 Hz) was accomplished during ambulation and rest. Velocity of animal movement was collected simultaneously using a locomotion tracking software (Neurotar, Helsinki, Finland). One field of view (FOV) was randomly selected within the boundaries of the hind limb area of S1 (left or right hemisphere, equally distributed across all animals), and was stored for further analysis.

### 2.6 2P image processing and extraction

Signal processing and data extraction were accomplished using a custom MATLAB pipeline as previously described (Lin et al., 2022). Image stacks of each file were imported as cubes (X and Y [pixels] and timepoints). For network and Δ*F*/*F* Ca^2+^ analysis, regions of interest (ROIs) of individual neurons were selected using adaptive thresholding (sensitivity at 0.35) to binarize potential ROIs followed by size (>60 pixel) and shape (0–0.9 eccentricity) filters to remove any undersized or irregularly shaped ROIs, as these likely represent imaging artifacts or cellular debris. All FOVs were checked for the presence of clear, morphologically distinct single neurons. The thresholded and filtered image was then used to extract raw GCaMP8f signal intensities across time (traces) for all ROIs in the FOV. Across all 2P Ca^2+^ data, we analyzed a total of 564 neurons from seven young males, 423 neurons from five aged males, 574 neurons from six young females, and 498 neurons from five aged females.

### 2.7 Network analysis

For each ROI, the raw trace underwent a continuous wavelet transform (CWT) using a Morse wavelet to extract power across frequencies (0.05–14 Hz). The peak magnitude within discrete frequency bands (i.e., 0.1–0.5, 0.5–1.0, 1.0–2.0 Hz, etc.) of the CWT spectrum was then determined for each frequency band across time, then binarized. An event was identified if the power was >2 times the standard deviation from the mean power, providing a dataset of ones (events) and zeroes (non‐events) across time. Measures of active neurons were calculated by dividing the number of neurons with at least one event during ambulation in each FOV by the FOV's area in mm^2^. Binarized events were used to calculate a correlation coefficient (CC) for each pair of neurons across time. We initially selected a thresholded CC value of >0.4, allowing us to select for pairs of neurons with 40% coinciding activity (considered an active connection). Connectivity was determined as the number of active connections per mm^2^ and connection length was defined as the average distance (in μm) between each pair. Network synchronicity was derived by calculating a ratio of the number of events divided by the total number of possible events for each neuron and is provided as a percentage of the time. To calculate the total number of possible events, we used the maximum of each frequency range. For instance, to analyze 1–2 Hz range, we multiply 2 Hz by the length of the period (the animal was either ambulating or resting) to yield the number of total possible events per neuron (e.g., 2 Hz × 45 s = 90 total possible events). We report network measures collected from 23 GCaMP8f‐treated animals (young male *n* = 7, aged male *n* = 5, young female *n* = 6, aged female *n* = 5).

### 2.8 Δ*F*/*F* analysis

For ΔF/F analysis, the threshold for an event in each ROI was set to >2 times the standard deviation from the mean GCaMP8f intensity during resting (i.e., animal stationary). Once identified, Ca^2+^ transients were then characterized for measures of neuronal activity (# of events detected per second), area‐under‐curve, rise‐time, and decay‐time constants. Each trace was normalized to resting fluorescence values (%Δ*F*/*F*). One young female displayed values on measures of area‐under‐curve (Figure 5b) reported as outliers, we therefore report Δ*F*/*F* measures collected from 22 GCaMP8f‐treated animals (young male *n* = 7, aged male *n* = 5, young female *n* = 5, aged female *n* = 5).

### 2.9 Gait behavior

For comparison of gait across age, animals were recorded using a digital camera (3840 × 2160 pixels at 25 Hz) ambulating across a 3‐plane visualization walking task built in‐house (Lin et al., 2022). Animals were recorded ambulating across four surfaces (3 mm, 4 mm, and 5 mm plastic mesh; and flat control). Animals that stopped midway through the task were placed at the beginning of the corridor and re‐tested until they completed at least four consistent, consecutive steps per surface. We report gait measures collected from 31 GCaMP8f‐treated animals (young male *n* = 8, aged male *n* = 6, young female *n* = 10, aged female *n* = 7).

### 2.10 Quantification of gait parameters

We used previously published methods (Lin et al., 2022) to quantify gait behavior using a reduced corridor width (4.2 cm) in order to accommodate for the smaller animals used here. Briefly, we analyzed the portion of videos with four consistent, consecutive steps in FIJI (ImageJ2 v2.3.0/1.53q) to extract the X and Y position of each paw across time. Measures of locomotor stability were calculated as previously described in Lin et al. (2022) including: ambulation velocity (cm/sec), deviance from center index—the sum of all paw distances (in cm) from the center of the corridor divided by four steps, paw precision index—average of r^2^ values derived from each paw's linear regression across four steps, and total stride deviance index—standard deviation of absolute distances between step‐to‐step paw placements for each paw. Additionally, stride length and a stride time deviation were calculated as follows: stride length—average distance (in cm) between step‐to‐step paw placement for each paw across four steps; stride time deviation—the standard deviation of time (in seconds) between step‐to‐step paw placements for each paw across four steps.

### 2.11 Experimental design and statistical analysis

Due to the complexity of the experiment design which includes young and aged animals undergoing a chronic craniotomy, training on the Neurotar HomeCage environment, 2P imaging of ambulating animals, and collection of grip strength and gait measures, some animals do not have complete datasets for every measure (see Table 1). All statistical analyses were performed using GraphPad Prism 9 (GraphPad Software Inc., San Diego, CA). All datasets were tested for significance using either a two‐way or a three‐way ANOVA (with or without repeated measures [RM]). For all measures of gait (Figure 6), one surface (4‐mm) was missing from one young female due to a computer recording error; thus, a mixed effects (REML) analysis was used. Significance for all measures was defined as *p* < 0.05. All data are presented as means ± SE of the mean. For all graphs with *n* < 10, we included individual data points.

**TABLE 1:** Number of subjects per group reported for each measure.

	Males		Females	
	Young	Aged	Young	Aged
Grip strength	8	9	11	8
Neuronal network dynamics	7	5	6	5
Δ*F*/*F* neuronal Ca^2+^ dynamics	7	5	5	5
Gait behavior	8	6	10	7

## 3 RESULTS

### 3.1 Grip strength testing

**FIGURE 1:**
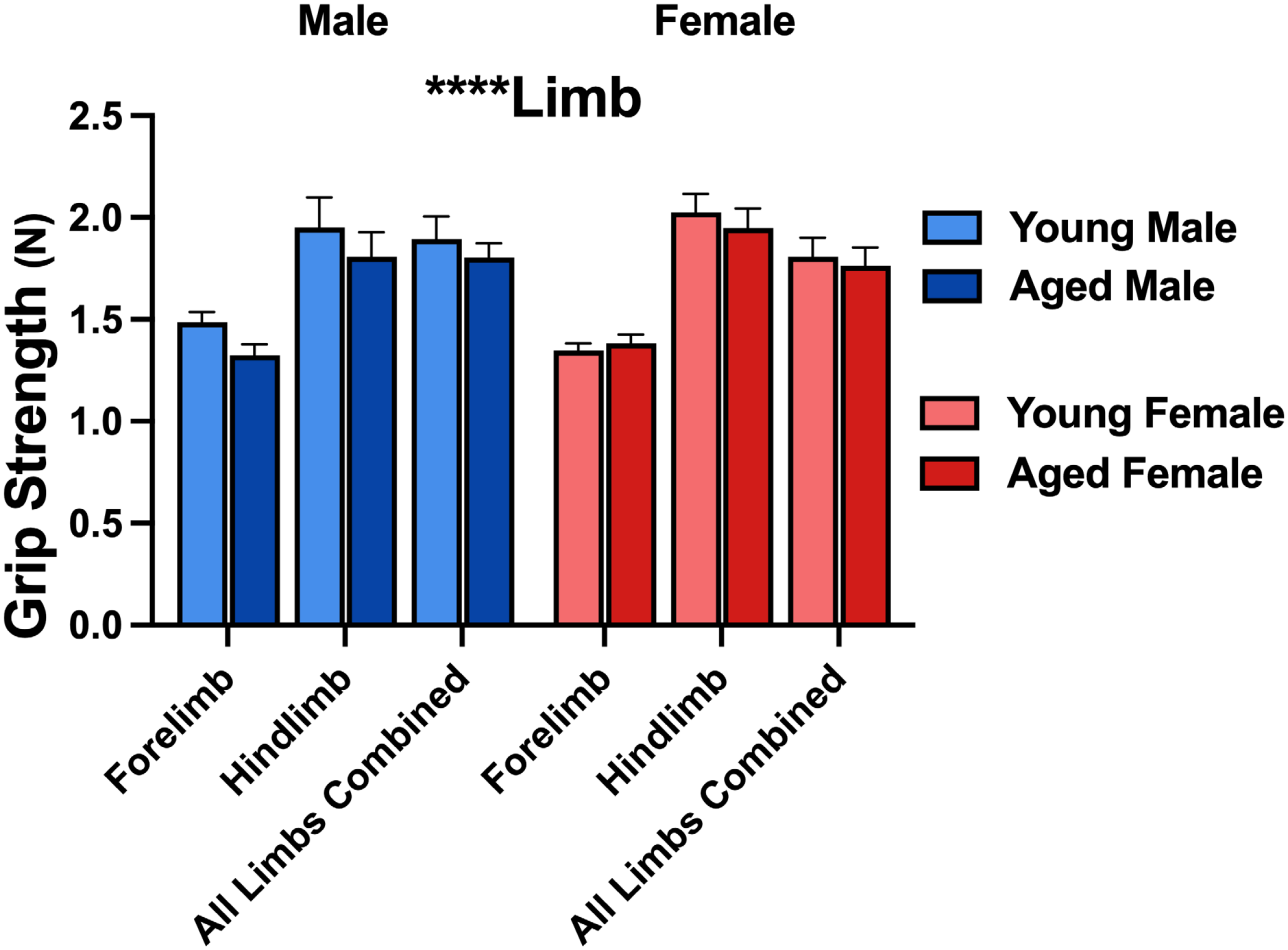
Grip strength in young and aged mice. Measures of grip strength for forelimb, hind limb, and all limbs combined were obtained in young male (*n* = 8), aged male (*n* = 9), young female (*n* = 11), and aged female (*n* = 8) C57BL/6J mice. While a limb effect (i.e., forelimb vs. hind limb) was noted, no sex‐ or age‐associated changes were present. Data are expressed in Newtons (*N*). Main effect of limb was identified with *p* < 0.0001.

Given that prior literature has shown an increase in sarcopenia with age in both preclinical and clinical models (Shavlakadze et al., 2010; Walston, 2012), and that we were quantifying measures of ambulation, it was important to test for sex or age differences in the grip strength (in Newtons) of forelimbs and hindlimbs for each animal in the study. Results indicated no significant differences in grip strength across age or sex, but a significant limb difference was noted (Figure 1; 3‐way RM ANOVA; *F*_2,64_ = 47.25; *p* < 0.0001), suggesting that forelimbs and hindlimbs contribute differentially to overall limb strength. We specifically did not normalize these data to body weight and report the numbers in Newtons as the females (average young weight: 23.3 ± 0.4 grams; average aged weight: 32.5 ± 1.4 grams) were much smaller (data not shown; 2‐way ANOVA; *F*_1,32_ = 29.0, *p* < 0.0001) than males (average young weight: 30.0 ± 0.7 grams; average aged weight: 42.2 ± 2.6 grams), which would have artificially inflated their measures of grip strength compared to male, if normalized to body weight (Bonetto et al., 2015).

### 3.2 Neuronal network dynamics

**FIGURE 2:**
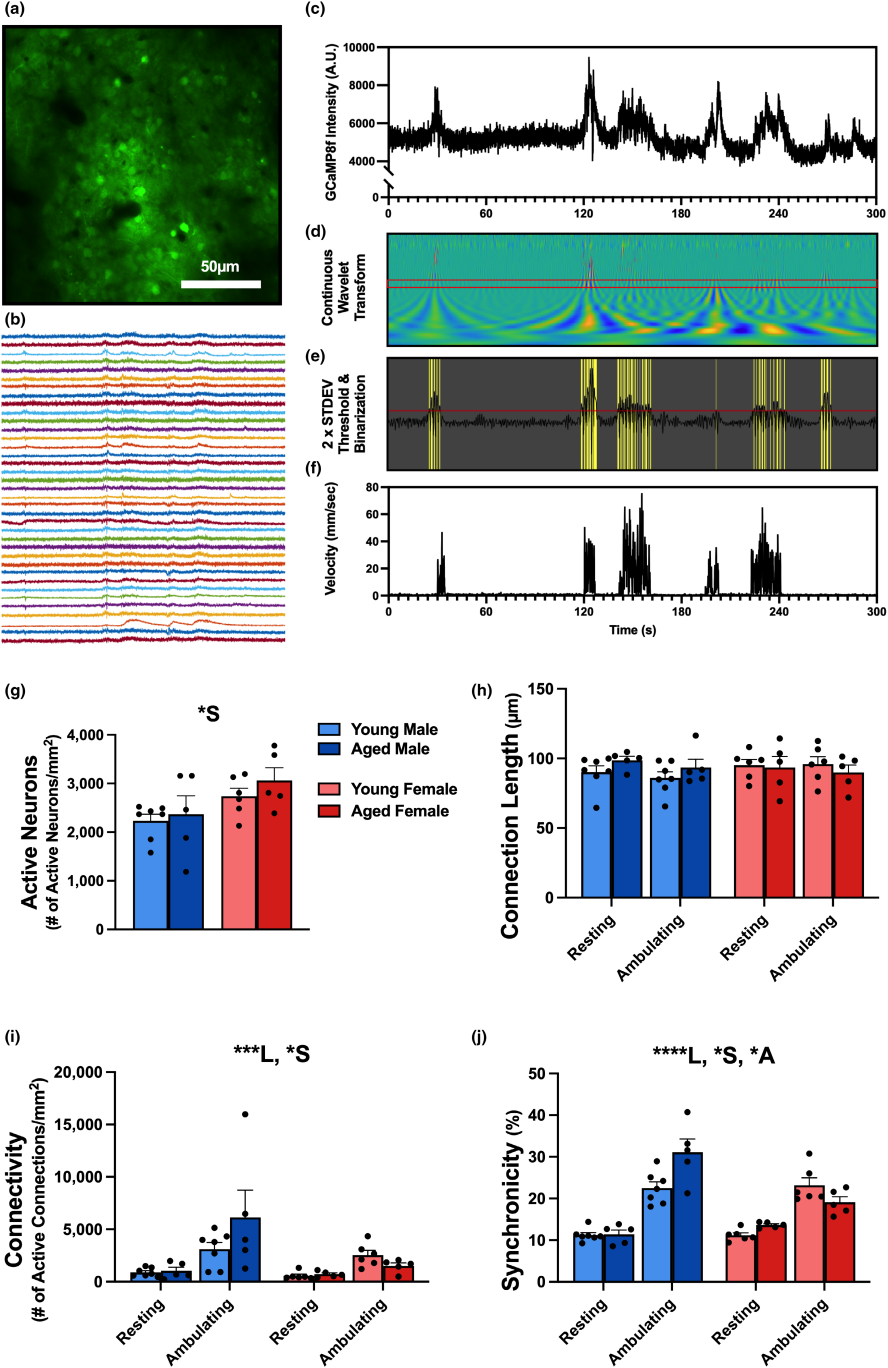
S1 neuronal network dynamics in young and aged mice on flat surface. (a) Individual GCaMP8f‐positive neurons are distinguishable in S1 in an aged male during ambulation and rest across a flat surface. Dark circular areas reflect penetrating blood vessels. (b) Representative GCaMP8f intensity traces from ROIs identified in panel (a) during periods of rest and ambulation. Scale bar represents 30 secs. (c) Individual raw GCaMP8f signals during rest and ambulation underwent a continuous wavelet transform (d) and were then thresholded (red line) and binarized (yellow vertical lines) (e) to extract events across multiple frequencies. (f) Corresponding animal's ambulation velocity trace across time. Measures of network communication (g–j) were obtained in young male (*n* = 7), aged male (*n* = 5), young female (*n* = 6), and aged female (*n* = 5) C57BL/6J mice and here, we report measures in the 0.1–2 Hz frequency domain. A main effect of sex was detected on measures of active neurons (g), connectivity (i), and synchronicity (j). A significant locomotion effect (ambulating vs. resting) was noted for measures of connectivity (i) and synchronicity (j). Additionally, a main effect of age was reported for measures of synchronicity (j) with age males having a significantly greater synchronicity compared to young males. No significant changes were detected on measures of connection length (*p* > 0.05). Main effects with *p* < 0.05, *p* < 0.001, and *p* < 0.0001 are represented with *, ***, and ****, respectively. Main effects of age, locomotion, and sex are represented with A, L, and S, respectively.

**FIGURE 3:**
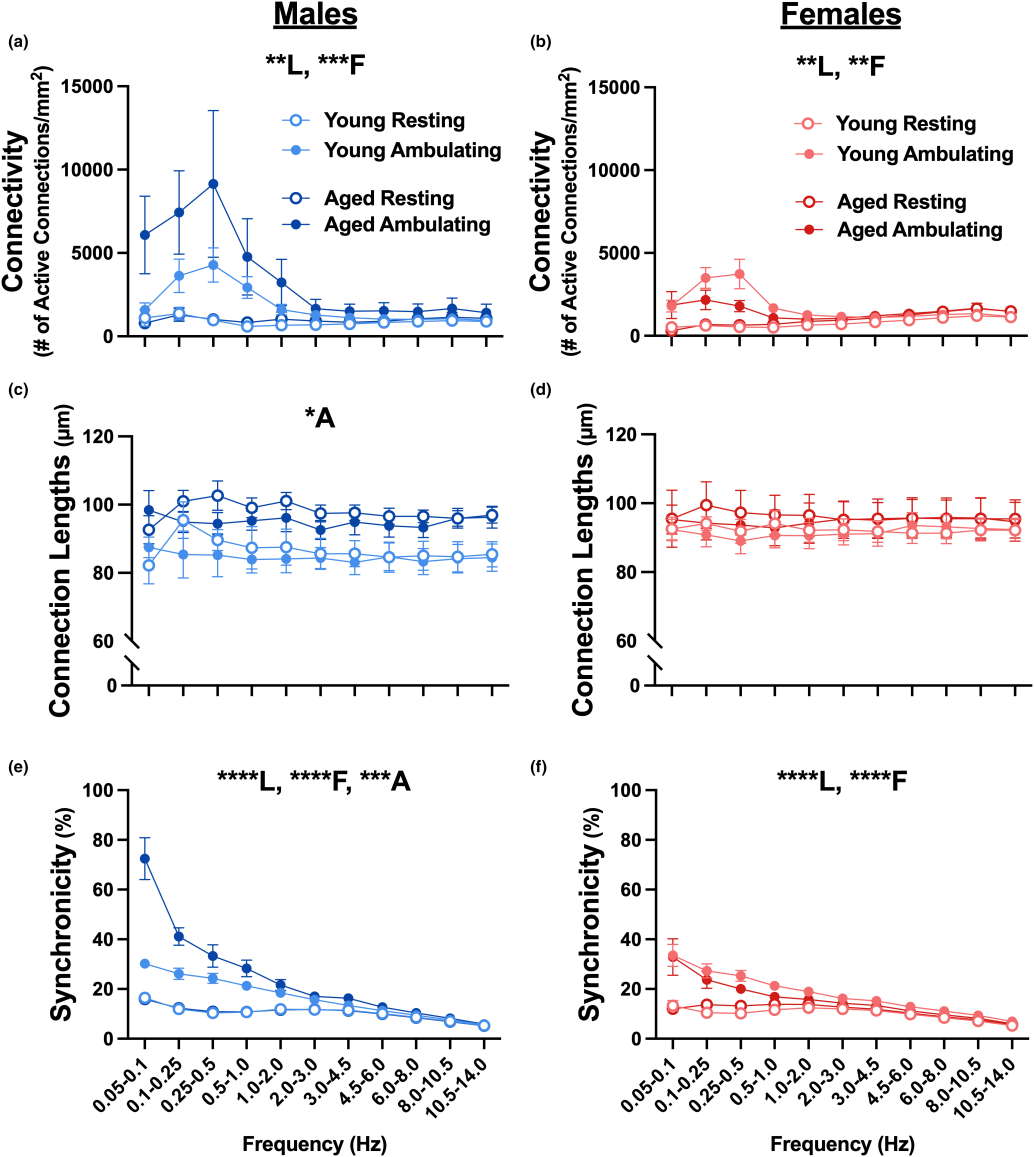
Neuronal network dynamics across frequency on flat surface. Measures of network communication were obtained in young male (*n* = 7), aged male (*n* = 5), young female (*n* = 6), and aged female (*n* = 5) C57BL/6J mice across multiple frequency domains during ambulation and rest. Main effects of locomotion status and frequency were detected on measures of connectivity (a, b) and synchronicity (e, f) in both males and females. A main effect of frequency was noted for measures of synchronicity. Interestingly, we note a significant effect of age on measures of connection length (c) and synchronicity (e) in males only, where aged males had greater lengths of connections and synchronicity than young males. No significant changes were detected on measures of connection length in female mice (d; *p* > 0.05). Main effects with *p* < 0.05, *p* < 0.01, *p* < 0.001, and *p* < 0.0001 are represented with *, **, ***, and ****, respectively. Main effects of age, locomotion, and frequency are represented with A, L, and F, respectively.

We used previously published methods (Lin et al., 2022) to acquire, extract, and binarize GCaMP8f events during ambulation on a flat surface (Figure 2a–e). These data were obtained concomitantly to the velocity trace (Figure 2f) from the animal's recorded ambulation on the Neurotar Mobile HomeCage (Figure S1). Briefly, using the CWT to extract power of GCaMP8f intensity across frequencies, we note here significant differences across sex, age, and locomotion status in the 0.1–2 Hz range. This frequency range was selected based on prior literature and data presented in Figure 3 and Figure S2. A significant main effect of sex was identified for active neuron numbers (Figure 2g; 2‐way RM ANOVA, *F*_1,19_ = 6.53, *p* = 0.0193), connectivity (Figure 2i; 3‐way RM ANOVA, *F*_1,19_ = 4.717, *p* = 0.0427), and synchronicity (Figure 2j; 3‐way RM ANOVA, *F*_1,19_ = 6.903, *p* = 0.0166) engaged during movement. Additionally, a main effect of locomotion status was noted with increased measures of connectivity (Figure 2i; 3‐way RM ANOVA, *F*_1,19_ = 20.55, *p* = 0.0002) and synchronicity (Figure 2j; 3‐way RM ANOVA, *F*_1,19_ = 103.8, *p* < 0.0001), showing that these values are increased during ambulation. Interestingly, a significant effect of age was noted for measures of synchronicity (Figure 2j; 3‐way RM ANOVA, *F*_1,19_ = 4.194, *p* = 0.05), where aged males showed increased synchronicity during ambulation only. From these measures (Figure 2g–j) obtained across sex or age, none showed significant differences during resting, suggesting neuronal network changes at rest are likely subtle.

One advantage of using the CWT to extract GCaMP8f signals is that it allows for extraction of power across multiple frequency domains (0.05–14 Hz) and investigation of the alignment between ambulatory behavior and network activity across these frequencies (Figure S2). Here, we show a main effect of locomotion (Figure 3a; 3‐way RM ANOVA, *F*_1,20_ = 9.149, *p* = 0.0067; Figure 3b; 3‐way RM ANOVA, *F*_1,18_ = 13.21, *p* = 0.0019) and frequency (Figure 3a; 3‐way RM ANOVA, *F*_1.708, 34.17_ = 9.609, *p* = 0.0008; Figure 3b; 3‐way RM ANOVA, *F*_1.879, 33.82_ = 6.057, *p* = 0.0065) on measures of connectivity, showing that during ambulation, there is an increase in connectivity in the lower frequency domains (0.1–2 Hz). Additionally, a main effect of locomotion (Figure 3e; 3‐way RM ANOVA, *F*_1,20_ = 105.9, *p* < 0.0001; Figure 3f; 3‐way RM ANOVA, *F*_1,18_ = 42.14, *p* < 0.0001), frequency (Figure 3e; 3‐way RM ANOVA, *F*_1.979, 39.58_ = 128.6, *p* < 0.0001; Figure 3f; 3‐way RM ANOVA, *F*_1.238, 22.28_ = 53.3, *p* < 0.0001), and age, carried mostly by males (Figure 3e; 3‐way RM ANOVA, *F*_1,20_ = 15.25, *p* = 0.0009), was noted for measures of synchronicity. As previously reported in other brain regions, we show that irrespective of frequency, aged males display greater connection length (Figure 3c; 3‐way RM ANOVA, *F*_1,20_ = 7.841, *p* = 0.0111) in the activated network of S1, compared to young males. This result is in line with the concept that with aging, larger receptive field size as well as reduced place cell specificity are seen (Beauchet et al., 2019; David‐Jurgens et al., 2008; Mehta et al., 1997). Due to the nature of our statistical analysis (3‐way RM ANOVAs), we purposely focused on aging differences within each sex, and therefore, could not identify main effects of sex for these CWT‐derived measures. It is interesting to note, however, that males appear to show greater connectivity and synchronicity compared to females, which was shown to be significant on the flat surface (Figure 2i,j).

**FIGURE 4:**
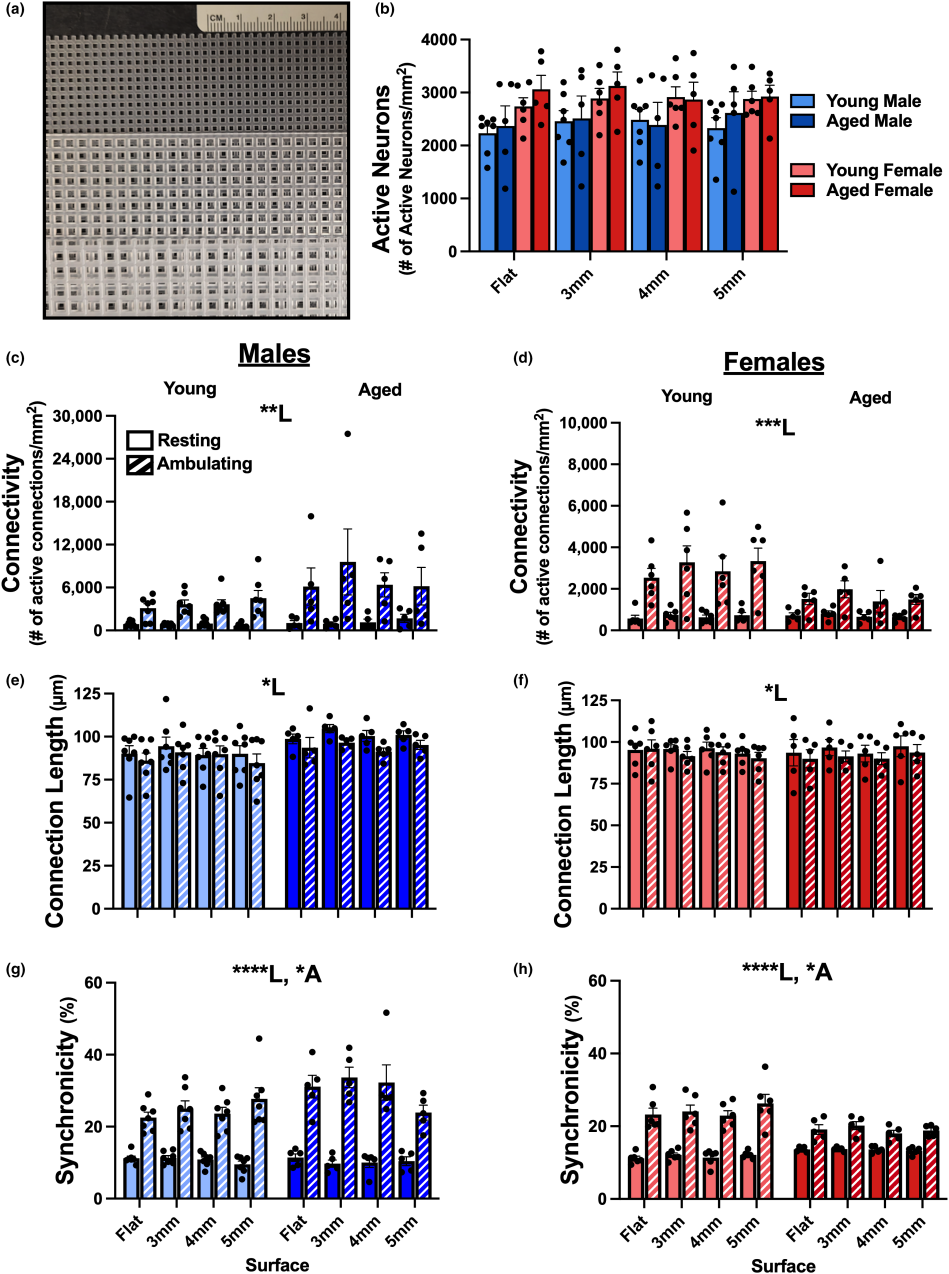
Neuronal network dynamics in young and aged mice across multiple surfaces. Measures of network communication were obtained in young male (*n* = 7), aged male (*n* = 5), young female (*n* = 6), and aged female (*n* = 5) C57BL/6J mice during ambulation and rest across multiple surfaces (a; 3‐mm, 4‐mm, and 5‐mm plastic mesh), and here, we report measures in the 0.1–2 Hz frequency domain. A main effect of locomotion status was detected on measures of connectivity (c, d), connection length (e, f), and synchronicity (g, h) in both males and females. Additionally, we note a significant effect of age on measures of synchronicity in both male and female mice (g, h), where aged males had greater synchronicity than young males, while aged females had decreased synchronicity compared to young females. No significant changes were detected on measures of active neurons (b) across sex, age, or surface (*p* > 0.05). Main effects with *p* < 0.05, *p* < 0.01, *p* < 0.001, and *p* < 0.0001 are represented with *, **, ***, and ****, respectively. Main effects of age and locomotion are represented with A and L, respectively.

We used neuronal network analyses during ambulation across different substrates as a proxy to measures of tactile discrimination and proprioception. Specifically, a flat surface, or plastic mesh with 3‐mm, 4‐mm, or 5‐mm spacing (see Figure 4a), were used to test for differences in measures of neuronal network communication. While Figure 4b shows that there were no differences in the number of active neurons engaged during ambulation across surface, age, and sex at any time, our results show a main effect of locomotion on all measures (Figure 4c–f), with increases in activity during ambulation irrespective of age or sex. For measures of synchronicity in both sexes, again, we showed a main effect of locomotion; however, there was also a main effect of age, where males appeared to display increases in synchronicity with age (Figure 4g; 3‐way RM ANOVA; *F*_1,10_ = 5.565, *p* = 0.04), while females appeared to show decreases in synchronicity with age (Figure 4h; 3‐way RM ANOVA; *F*_1,9_ = 5.41, *p* = 0.045). These effects only appear during periods of ambulation as *post hoc* analyses revealed that none of the measures presented in Figure 4 were significant during the resting period.

**FIGURE 5:**
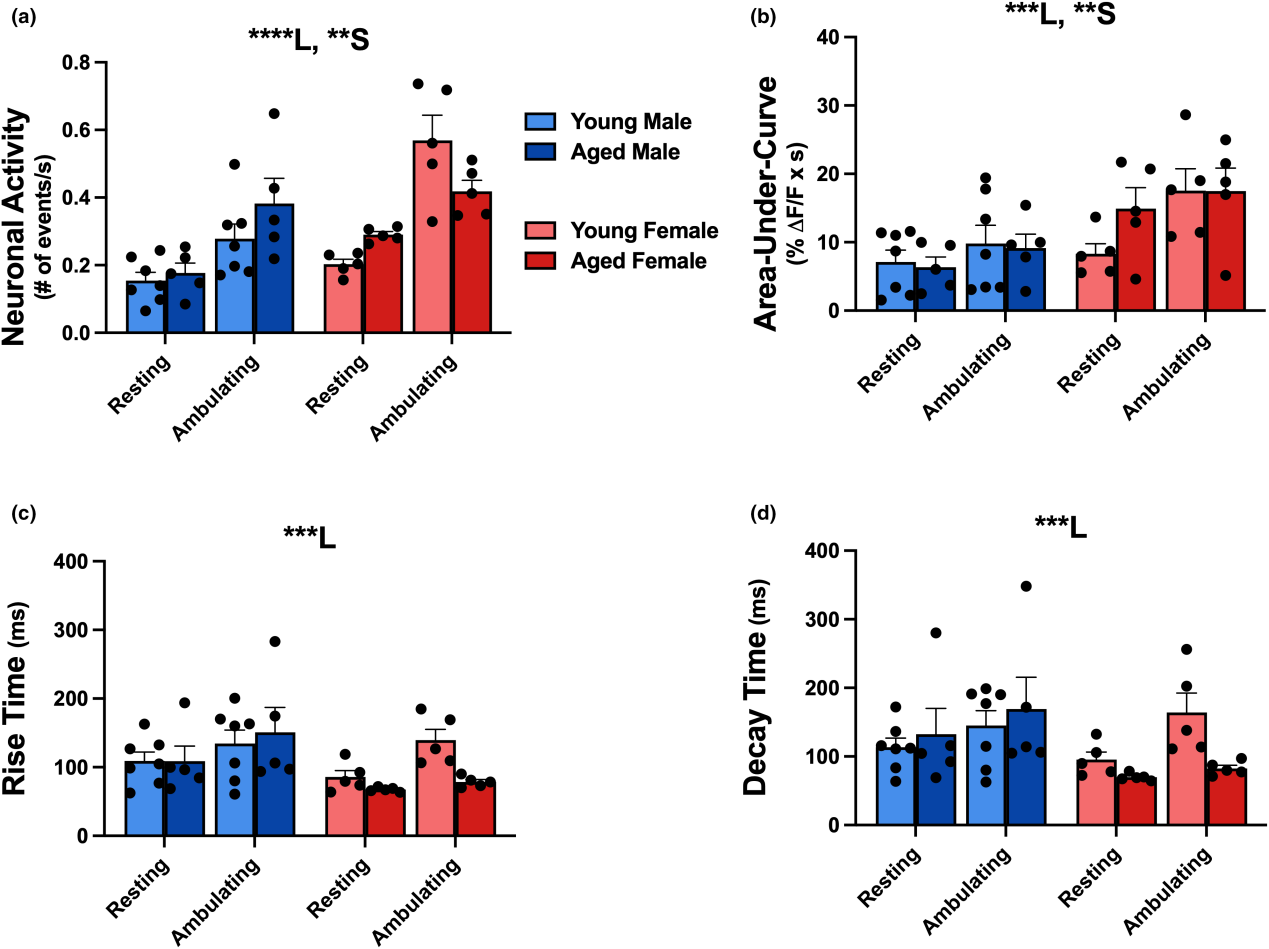
Single‐cell (Δ*F*/*F*) neuronal Ca^2+^ dynamics on flat surface. Measures of single‐cell (Δ*F*/*F*) neuronal Ca^2+^ dynamics were obtained in young male (*n* = 7), aged male (*n* = 5), young female (*n* = 5), and aged female (*n* = 5) C57BL/6J mice during ambulation and rest across a flat surface. We report a main effect of locomotion status for measures of neuronal Ca^2+^ transients (a), area‐under‐curve (b), rise time (c), and decay time (d). Additionally, we report a main effect of sex for measures of neuronal activity (a) and area‐under‐curve (b). Main effects with *p* < 0.01, *p* < 0.001, and *p* < 0.0001 are represented with **, ***, and ****, respectively. Main effects of locomotion and sex are represented with L and S, respectively.

While the CWT routine provided depth and clarity on analyses of neuronal Ca^2+^ dynamics across frequencies and also reported on network‐level measures of connectivity and synchronicity across hundreds of neurons, the binarization routine prevented us from addressing more traditional methods of Ca^2+^ transients, including measures of area‐under‐curve, rise‐time, and decay‐time constants of the Ca^2+^ events. For these reasons, and to address rigor and reproducibility, we also performed a Δ*F*/*F* analysis (Figure 5) of the same dataset that was used to extract measures using the CWT routine. Using a Δ*F*/*F* method to identify neuronal activity, we showed a main effect of sex and locomotion on the number of Ca^2+^ events identified per second. We chose to represent this data as events/s, as animals spend different amounts of time either resting or ambulating during the imaging session, and focused on the flat surface, as we found no prior main effect of surface in Figure 4. As with CWT measures, a main effect of locomotion was noted for measures of neuronal activity (Figure 5a; 3‐way RM ANOVA; *F*_1,18_ = 74.08, *p* < 0.0001), area‐under‐curve (Figure 5b; 3‐way RM ANOVA; *F*_1,18_ = 18.57, *p* = 0.0004), rise time (Figure 5c; 3‐way RM ANOVA; *F*_1,18_ = 21.95, *p* = 0.0002), and decay time (Figure 5d; 3‐way RM ANOVA; *F*_1,18_ = 17.88, *p* = 0.0005). We also saw a significant main effect of sex for measures of neuronal activity (Figure 5a; 3‐way RM ANOVA; *F*_1,18_ = 10.99, *p* = 0.0039) and area‐under‐curve (Figure 5b; 3‐way RM ANOVA; *F*_1,18_ = 7.877, *p* = 0.0117). The alignment between these two methods of analyses further translated to the directionality of the effect, where females showed an increase in the number of active neurons or events/s (Figures 2g and 5a) as compared to males, but also showed decreases in connectivity and synchronicity (Figure 2i,j) paired with increases in Ca^2+^ transient area‐under‐curve (Figure 5b).

### 3.3 Gait behavior testing

**FIGURE 6:**
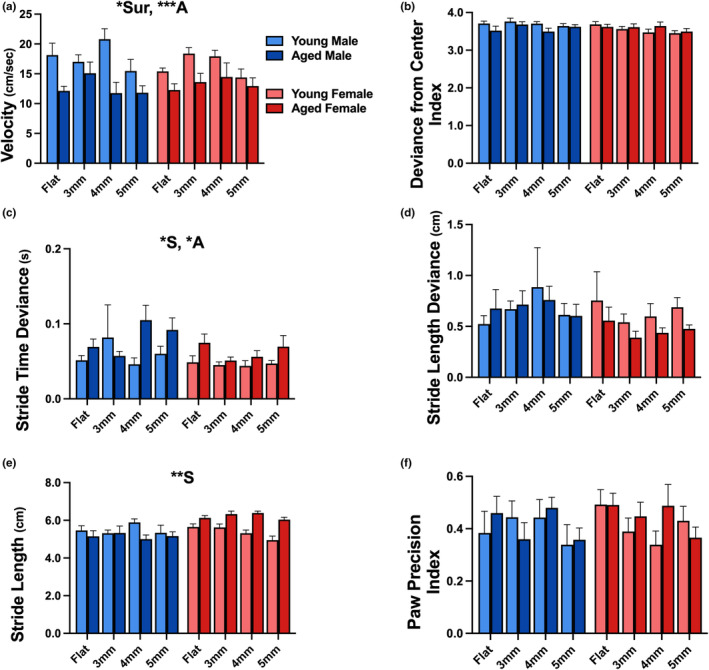
Gait behavior in young and aged mice across multiple surfaces. Measures of gait behavior were obtained in young male (*n* = 8), aged male (*n* = 6), young female (*n* = 10), and aged female (*n* = 7) C57BL/6J mice during ambulation across a flat (control), 3 mm, 4 mm, and 5 mm plastic mesh surface. A main effect of age was noted for measure of velocity (a) and stride time deviance (c), highlighting that aged animals ambulate slower and perhaps exhibit impaired gait rhythm or cadence. We report a main effect of sex for measures of stride time deviance (c) and stride length (e). Interestingly, we report a main effect of surface for measures of velocity (a). No main effects were seen for measures of deviance from center (b), stride length deviance (d), and paw precision (f). Main effects with *p* < 0.05, *p* < 0.01, and *p* < 0.001 are represented with *, **, and ***, respectively. Main effects of age, sex, and surface are represented with A, S, and Sur, respectively.

Following 2P neuronal Ca^2+^ imaging, we used a 3‐plane visualization walking task (Lin et al., 2022) to obtain measures of gait coordination, velocity, and stride across age, sex, and surface. A main effect of age was seen for measures of velocity (Figure 6a; 3‐way RM ANOVA; *F*_1,27_ = 16.75, *p* = 0.0003) and stride time deviance (Figures 6c and 3‐way RM ANOVA; *F*_1,27_ = 6.162, *p* = 0.0196), highlighting that aged animals ambulate slower and exhibit impaired gait rhythm. Furthermore, a main effect of sex was noted for measures of stride time deviance (Figure 6c; 3‐way RM ANOVA; *F*_1,27_ = 4.334, *p* = 0.0470) and stride length (Figure 6e; 3‐way RM ANOVA; *F*_1,27_ = 9.445, *p* = 0.0048), where females show less deviance and slight increases in stride length compared to males. We detected a main effect of surface on measures of velocity (Figure 6a; 3‐way RM ANOVA; *F*_3,80_ = 3.915, *p* = 0.0116), suggesting this behavioral task is able to identify surfaces that present with greater challenges for the aged animals.

## 4 DISCUSSION

This study examined neuronal Ca^2+^ dynamics across sex in young (4 months) and aged (22 months) ambulating C57BL/6J mice using two methods (CWT and Δ*F*/*F*). The work includes over 2000 active neurons imaged across four groups (young male, aged male, young female, aged female), and reports on changes in layers 2/3 of S1 while investigating the relationship between Ca^2+^ dynamics and gait dysregulation. We specifically tested the hypothesis that age‐ and sex‐dependent alterations in S1 neuronal networks are present in the awake, ambulating mouse during behavioral engagement when measured across tens of neurons communicating in local circuits.

Our results align well with previously published work using different methods in awake animals showing cortical signals spontaneously oscillating in the lower delta wave range (Llano et al., 2021; Miller et al., 2014; Poulet et al., 2012; Sun & Dan, 2009; Zhang et al., 2021). To our knowledge, this is likely one of the first analyses of individual neurons showing activity in lower frequency domains (0.1–2 Hz) during ambulation and rest in S1 of young and aged animals. Importantly, within these frequencies, we report on significant, albeit small, age‐dependent increases in neuronal synchronicity during ambulation in males (Figures 2j, 3e and 4g). In some cases, synchronicity reached nearly 70% in the aged male, which might be reflective of poor single‐cell encoding, but depending on the brain regions, it is unclear whether increases or decreases in synchrony are beneficial. Many studies have examined cortical synchrony either within, or across large brain regions using electroencephalogram (EEG) techniques, where neuronal activity and synchrony report on specific behavioral tasks, and are reflective of engagement and activation (Uhlhaas et al., 2009). Increases in synchrony across spatially distinct brain regions are often associated with improved memory performance, specifically in the context of memory encoding where hippocampal connections to the prefrontal cortex are investigated (Jones & Wilson, 2005; O'Neill et al., 2013; Stern et al., 2004). Interestingly, with human aging, investigations using EEG and diffusor tensor imaging have also described network synchrony as a potential mechanism for reduced cognitive performance (Hinault et al., 2021).

However, within a single brain region, such as S1, prior work has shown the presence of network desynchronization during stimulation (whisker or visual stimuli) compared to quiet wakefulness (Arroyo et al., 2018; Khateb et al., 2021; Poulet et al., 2012), highlighting that while the neuronal network may be synchronous at rest, neurons also exhibit complex individual patterns during activation. This is unsurprising, as it is well‐known that sensory encoding occurs at the level of individual cells, where neurons are shown to have specificity and tuning to various stimuli (Mehta et al., 1997; O'Keefe & Conway, 1978; Zong et al., 2022), likely participating in some form of desynchronization. Indeed, prior descriptions based on single‐ or few‐cell measures have shown behavior‐dependent changes in neuronal activity (Kang et al., 2010; Salinas et al., 2000; Sun & Dan, 2009; Wirtshafter & Disterhoft, 2022; Zhao et al., 2012, 2016). However, the use of single cell measures in these studies precludes analyses of the local network activity (Mohajerani et al., 2010), impeding measures of synchronicity. While much has been learned in the past 30 years from measures of single‐ and multi‐cell recording of the brain during activation, providing a clear picture that specific brain regions exhibit alterations in intrinsic excitability and spatial mapping specificity with age (Chang et al., 2005; Disterhoft & Oh, 2007; Hickmott & Dinse, 2013; Patrylo et al., 2007; Thome et al., 2016; Wilson et al., 2005), an important and addressable gap remains between single‐cell dynamics and local brain region activity.

Our measures of network communication report on somatosensory encoding during ambulation and rest within small cortical areas (176 × 176 μm) across hundreds of individual neurons. Importantly, and in alignment with others, we report on dynamic changes in neuronal network communication during ambulation compared to rest, including increases in connectivity and synchronicity. Of note, recent work has highlighted the importance of thalamic input on regulating cortical excitability (Poulet et al., 2012) and suggests that our measures may not necessarily be representative of within‐layer connectivity, but rather reflective of synchronization driven by thalamocortical and translaminar inputs. Specifically, neurons in layers 2/3 of S1 act as a local circuit which encodes information received from the thalamus and other movement‐associated cortical areas (Cichon & Gan, 2015), while neurons in layer 5 send outputs upward within cortical columns as well as to adjacent areas. Given that tactile discrimination and proprioception are important contributors to ambulation and that encoding of these inputs is layer‐specific, characterizing local circuit activity within layers 2/3 of S1 is crucial to our understanding of S1s feedback to other key brain regions that drive movement. Prior work in S1 has identified clear changes in neuronal activity both in vivo and ex vivo in response to stimulation, either peripherally or locally (Hickmott & Dinse, 2013; Lin et al., 2022; Popescu et al., 2021; Zhao et al., 2016). Many of these studies also highlight age‐dependent increases in intrinsic excitability of layer 2/3 and layer 5 neurons (Hickmott & Dinse, 2013; Popescu et al., 2021). Whether our reported increases in synchronicity are mediated by similar changes in intrinsic excitability or depend on the origin of the synaptic input (Zhao et al., 2016), remains to be determined. It is also unclear whether neuronal Ca^2+^ dysregulation that occurs with age is driving the network dysregulation presented here. This is because, in the current study, the Ca^2+^ transients are used as a proxy for binarization of the data and extraction of neuronal network variables. Nevertheless, we describe movement‐dependent increases in neuronal activity in layer 2/3 of S1, reinforcing the role of S1 in ambulatory‐related processes including tactile discrimination and proprioception.

Our lab has previously described alterations in gait performance with aging that reflect on dysregulation in neuronal networks using the F344 rat model of aging, where number of active neurons and connectivity were increased with aging (Lin et al., 2022). Here, our results in the ambulating C57BL/6J mouse highlight similar increases in connectivity with no changes in the number of active neurons. Additionally, while we saw large increases in measures of synchronicity with age in the behaving mouse, no alterations in synchronicity were noted in the anesthetized F344 rat. This is likely because the use of anesthesia has been shown to increase neuronal synchrony (Goltstein et al., 2015), possibly influencing our ability to detect age‐related effects in the anesthetized rat (i.e., ceiling effect). In both models of aging, however, we have now characterized ambulatory performance using the same 3‐plane visualization walking task, showing reduced ambulation speed. Here, we do not see the age‐dependent changes in deviance from center index as reported in the rat (Lin et al., 2022); instead, we note a significantly larger stride time deviance in aged, ambulating mice. This additional parameter of stride irregularity is reflective of impaired gait rhythm as seen in the clinic in the older adult (Kobsar et al., 2014).

Given that increased risk of falling is driven by gait dysregulation (Krauss et al., 2005), and that some studies show a decreased risk of falls in older females compared to older males (Aryee et al., 2017; Ek et al., 2019), it was necessary to consider sex as a biological variable in this study. We report decreased connectivity and synchronicity during ambulation in aged female mice compared to aged males. Importantly, we present evidence for increases in the number of active neurons and events/s in aged females, highlighting the possibility that these active neurons may be less synchronized. Perhaps this is representative of beneficial network‐level encoding traits in females that maintains distinct neuronal activity during a task, independent of nearby synchronicity. Furthermore, on measures of ambulatory performance, aged females show decreased stride time deviance and increased stride length compared to aged males. Given that smaller stride time deviance reflects better gait rhythm regularity (Kobsar et al., 2014), and that stride length is known to shorten with gait dysregulation (Duggan et al., 2017), these results strongly support the notion that decreased incidence and risk of falls in older females may be dependent on central changes in network encoding.

Our gait behavior results align reasonably well with human clinical data and the changes in network communication observed in this study may serve as a potential central mechanism contributing to these changes in ambulatory performance. Specifically, sex differences seen across network communication and gait may highlight a role of S1 in gait dysregulation, where neurons display more activity as well as less connectivity and synchronization in female mice, leading to enriched somatosensory encoding and improved gait. It is encouraging to note that using two separate approaches to characterize neuronal Ca^2+^ function (CWT and Δ*F*/*F*), we are able to detect similar results of sex‐ and locomotion‐dependent increases in neuronal activity (Figures 2g and 5a). This cross‐validation suggests the effects presented are robust and reliable. It is important to note that modalities of S1 (i.e., tactile discrimination or proprioception) were not addressed in the current study, limiting our understanding of S1's contribution to gait, and thus, future studies investigating which aspects of somatosensation are altered with age and sex are warranted.

Here, we tested the Ca^2+^ hypothesis of brain aging using a network analysis across hundreds of neurons in a new brain region during ambulation. It is important to consider that while GCaMP8f reports on Ca^2+^ dynamics, it was primarily used as a proxy for neuronal activity to infer on neuronal network communication. Although prior work indicates that changes in neuronal network dynamics across age are likely mediated by changes in calcium handling at the single‐cell level, here, we report little to no changes in single‐cell Ca^2+^ dynamics across age and sex in the awake, ambulating animal (Figure 5). Indeed, prior studies using biochemical, electrophysiological, molecular, and imaging techniques in the hippocampus, have identified a variety of mechanisms that are both sensitive to Ca^2+^ as well as aging. Initial work identified reductions of NMDA receptors with age and emphasized their role in neuronal excitability and synaptic plasticity (Clayton et al., 2002; Foster & Norris, 1997; Magnusson, 1998; Rosenzweig & Barnes, 2003). Additionally, age‐dependent increases in plasma membrane L‐type voltage‐gated Ca^2+^ channels (L‐VGCC) were identified as regulators of cellular excitability (Campbell et al., 1996; Moyer Jr. et al., 1992; Nunez‐Santana et al., 2014; Thibault et al., 2001; Thibault & Landfield, 1996). Lastly, ryanodine receptors (RyR) on the endoplasmic reticulum, responsible for Ca^2+^‐induced Ca^2+^ release have been shown to exhibit increased activity in animal models of aging and age‐matched controls (Gant et al., 2006; Kumar & Foster, 2005; Murchison & Griffith, 1999; Stutzmann et al., 2006). While we see sex‐dependent increases in Δ*F*/*F* measures of neuronal activity and area‐under‐curve in females compared to males, we report no significant age‐dependent Ca^2+^ alterations in the awake, ambulating mouse. It is likely, therefore, that changes in neuronal Ca^2+^ handling obtained under well‐controlled environments (i.e., slices, cultures) and conditions (i.e., anesthesia) do not translate well to the *in vigilo* condition. Clearly, future studies are needed to identify the sources of neuronal Ca^2+^ dysregulation with age, perhaps with a focus on local pharmacological intervention (NMDA receptors, L‐VGCC, and RyR) in the awake, behaving animal. Together, these future lines of investigation may provide insight on potential therapeutic targets that can help offset gait dysregulation and fall risk in the older adult population.

## AUTHOR CONTRIBUTIONS

Authors S.L.C. and O.T. conceptualized and wrote the manuscript. Authors S.L.C. and R‐L.L. acquired all data and analyzed results presented in the figures. Author R.‐L.L provided editorial feedback on the manuscript.

## CONFLICT OF INTEREST STATEMENT

The authors declare no competing financial interests.

## Supporting information


Figures S1–S2

## Data Availability

The datasets generated during and/or analyzed during the current study are available from the corresponding authors on reasonable request. The authors will make all custom codes and algorithms used to extract data accessible upon request.
