# Supplementary material for: Age‐ and sex‐dependent alterations in primary somatosensory cortex neuronal calcium network dynamics during locomotion
Source: Aging Cell. 2023 Jun 3;22(8):e13898. doi: 10.1111/acel.13898 (PMC10410056; doi:10.1111/acel.13898)
Supplement: Supplementary file 1 — Figures S1–S2 [file ACEL-22-e13898-s001.pdf]

Supplementary Figures

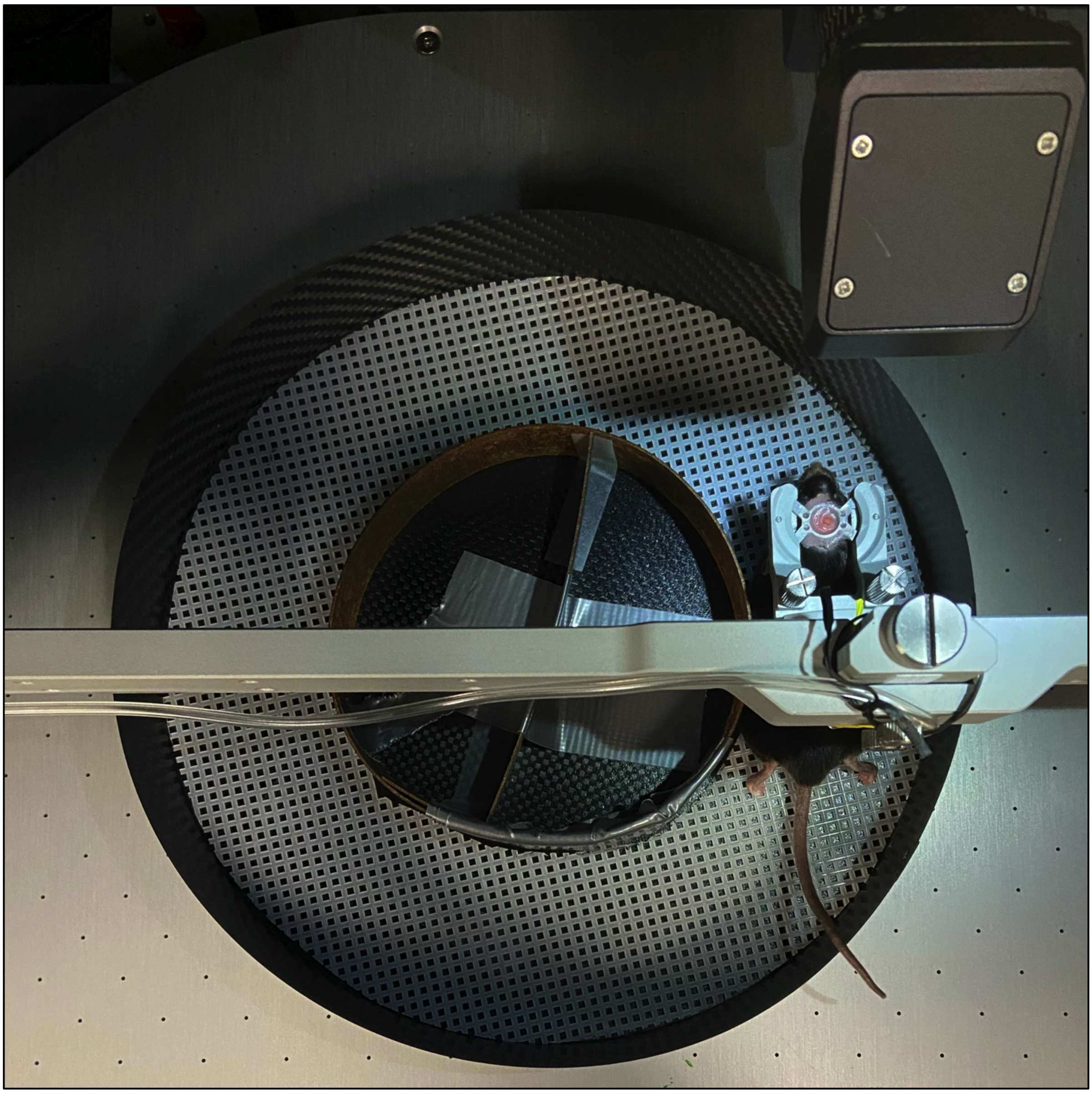

Supplemental Figure S1: Mouse on top of a plastic mesh surface (3-mm spacing) within the Neurotar Mobile HomeCage Large.

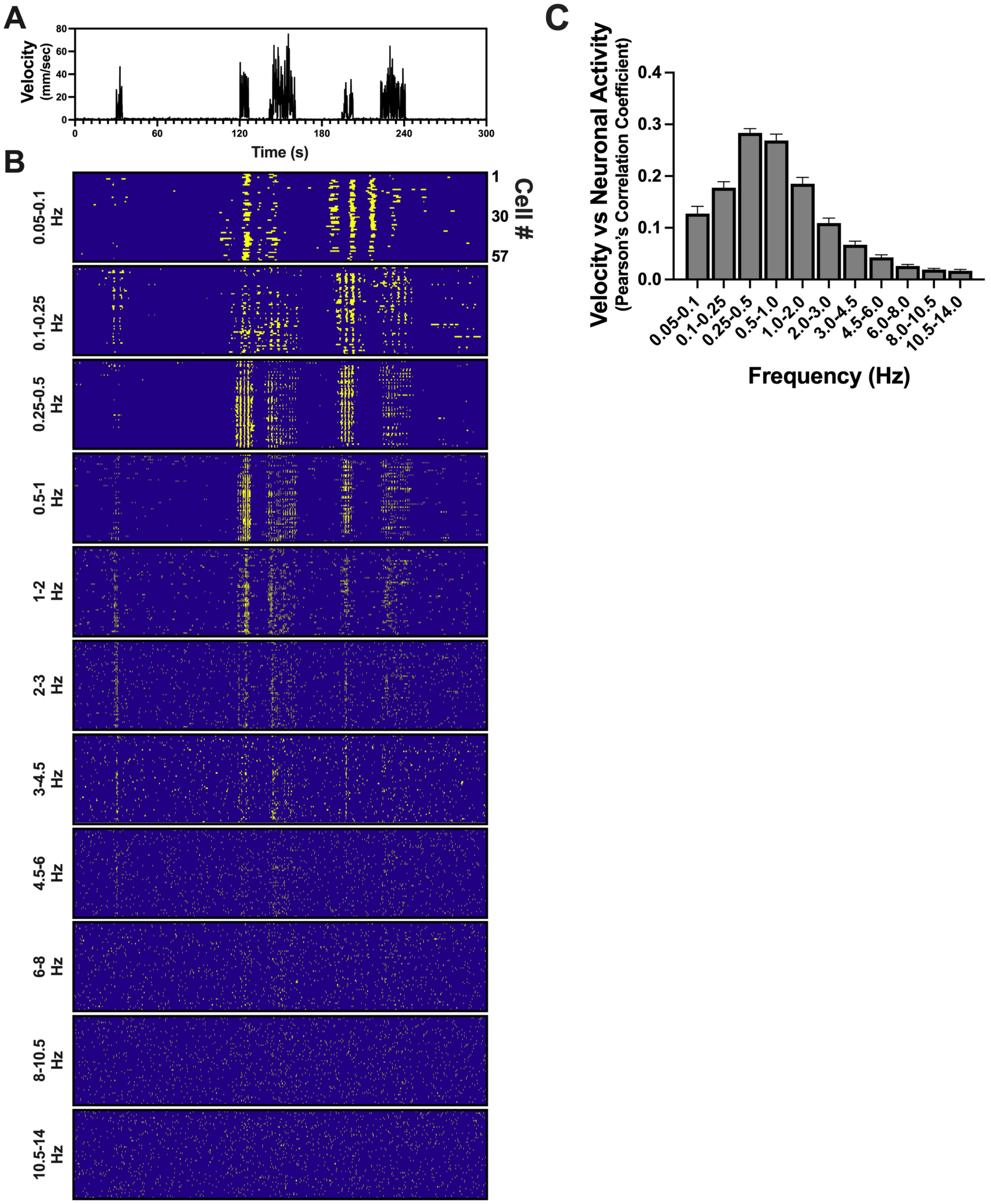

Supplemental Figure S2: Alignment of velocity with neuronal Ca^{2+} events across multiple frequency domains

(**A**) Representative velocity trace of aged male walking across a flat surface. (**B**) Corresponding raster maps of individual neuronal Ca^{2+} events across time (where each Y-axis value represents an individual neuron) derived using the CWT routine across multiple frequency domains. (**C**) Plot of correlation coeffects between velocity and neuronal activity across multiple

frequencies, highlighting that neuronal activity in lower frequencies of 0.1-2 Hz is clearly aligned with ambulatory behavior.
